# Noncoding RNAs in skeletal development and disorders

**DOI:** 10.1186/s40659-024-00497-y

**Published:** 2024-04-22

**Authors:** Qing Yao, Tailin He, Jian-You Liao, Rongdong Liao, Xiaohao Wu, Lijun Lin, Guozhi Xiao

**Affiliations:** 1 Department of Biochemistry, School of Medicine, Shenzhen Key Laboratory of Cell Microenvironment, Guangdong Provincial Key Laboratory of Cell Microenvironment and Disease Research, Southern University of Science and Technology, Shenzhen, 518055 China; 2 Guangdong Provincial Key Laboratory of Malignant Tumor Epigenetics and Gene Regulation, Sun Yat-Sen Memorial Hospital, Sun Yat-Sen University, Guangzhou, 510120 China; 3 Medical Research Center, Sun Yat-Sen Memorial Hospital, Sun Yat-Sen University, Guangzhou, 510120 China; 4 Department of Orthopedics, Zhujiang Hospital, Southern Medical University, Guangzhou, 510280 China

## Abstract

Protein-encoding genes only constitute less than 2% of total human genomic sequences, and 98% of genetic information was previously referred to as “junk DNA”. Meanwhile, non-coding RNAs (ncRNAs) consist of approximately 60% of the transcriptional output of human cells. Thousands of ncRNAs have been identified in recent decades, and their essential roles in the regulation of gene expression in diverse cellular pathways associated with fundamental cell processes, including proliferation, differentiation, apoptosis, and metabolism, have been extensively investigated. Furthermore, the gene regulation networks they form modulate gene expression in normal development and under pathological conditions. In this review, we integrate current information about the classification, biogenesis, and function of ncRNAs and how these ncRNAs support skeletal development through their regulation of critical genes and signaling pathways in vivo. We also summarize the updated knowledge of ncRNAs involved in common skeletal diseases and disorders, including but not limited to osteoporosis, osteoarthritis, rheumatoid arthritis, scoliosis, and intervertebral disc degeneration, by highlighting their roles established from in vivo, in vitro, and ex vivo studies.

## Introduction

Out of 2.85 billion nucleotides of the human genome, there are only 20,000–25,000 protein-encoding genes, which constitute less than 2% of total human genomic sequences [1]. The rest 98% of the genome was referred to as non-coding DNA, also known as “junk DNA”. The discovery of the central dogma of molecular biology states that genetic information is transcribed from DNA to RNAs and translated from messenger RNAs (mRNAs) to proteins, which facilitate all the biochemical reactions and cellular activities in cells [2]. The roles of functional RNAs, including mRNA, ribosomal RNA (rRNA), and transfer RNA (tRNA), were first identified in the process of protein synthesis. While mRNAs, which carry the genomic information for protein-encoding, are referred to as coding RNAs, non-coding RNAs (ncRNAs), such as rRNA and tRNA, with biological functions rather than encoding proteins have been known for more than sixty years [3].

In recent two decades, except for rRNA and tRNA, other functional ncRNAs, such as imprinted small nucleolar RNAs (snoRNAs), microRNAs (miRNAs), circular RNAs (circRNAs) and long non-coding RNAs (lncRNAs), have been identified and investigated. The first discoveries of small ncRNAs identified lineage defective 4 (lin-4) and lethal 7 (let-7) in *C.elegans.* They were reported decades ago, demonstrating that some ncRNAs are evolutionarily conserved and essential for development [4, 5]. With increasing numbers and varieties of ncRNAs identified, it became clear that functional products from the genome are not limited to proteins but also include functional ncRNAs. Some ncRNAs, such as miRNAs, snoRNAs, and circRNAs, are conserved across species, while others, including lncRNAs, normally lack evolutionary conservation. Functions of ncRNAs are various, including modifications of rRNAs, regulations of mRNA splicing and editing, altering the stability and translation of mRNAs, modulating chromatin functions, etc [6–9]. Many of these functions of ncRNAs ultimately affect gene expression in diverse cellular pathways and networks, leading to substantial alterations in fundamental cell processes, including cell proliferation, differentiation, apoptosis, and metabolism. Many ncRNAs have been confirmed to play important regulatory roles regarding gene expression in physiological processes and pathological conditions. MiRNAs, lncRNAs, circRNAs, and snoRNAs are involved in organelle development, stem cell differentiation, aging, tumorigenesis, cancers, metabolic disorders, etc. Some ncRNAs were identified as potential diagnostic biomarkers and therapeutic targets in cancers or other diseases.

The mammalian skeleton serves as a rigid internal frame of the body for supporting and protecting the soft internal organs, providing movement abilities. It is the critical site for blood cell formation. Skeletogenesis originates from migration and condensation of mesenchymal cells. The mesenchymal cells can directly differentiate into osteoblasts to form bone *via* a process referred to as intramembranous bone formation or into chondrocytes to form cartilage through endochondral ossification [10]. The skeletal system consists of bone, cartilage, joints, bone marrow, tendons, and ligaments. Dysregulation of gene expression during the development of the skeletal system leads to failure of bone formation, cartilage defects, mis-patterning of the skeleton, abnormalities of joint formation, etc. Loss of homeostasis, inappropriate external force, genetic defects, aging, and other factors may lead to skeletal disorders, including osteoporosis, osteoarthritis, rheumatoid arthritis, scoliosis, bone fracture, growth plate injuries, and intervertebral disc degeneration.

NcRNAs have been known to regulate the development of the nervous system, immune system, cardiovascular system, and other systems. In addition, accumulating evidence reveals that miRNAs play crucial roles in regulating key processes in skeletal development and are involved in osteoporosis, osteoarthritis, intervertebral disc degeneration, etc. In recent years, lncRNAs and circRNAs have also emerged as critical regulators of gene expression in bone and cartilage development and skeletal diseases. Interestingly, most of these regulatory ncRNAs are transcribed by RNA polymerase II (Pol II), whereas structural ncRNAs, like snRNAs, snoRNAs, and tRNAs, are primarily transcribed by polymerase III (Pol III). In this review, we integrate current information about the classification, biogenesis, and function of Pol II transcribed regulatory ncRNAs and how these ncRNAs support skeletal development through their regulation of key genes, signaling pathways, and networks in the skeleton. We also summarize the updated knowledge of ncRNAs involved in common skeletal diseases by highlighting the related in vivo studies and examining the evidence for *ex-vivo* and in vitro studies as well.

### Classification, biogenesis, and function of ncRNAs

Though protein-coding genes constitute only around 2% of the human genome sequences, modern transcriptome sequencing technology has revealed that at least 90% of the genome is actively transcribed [11, 12]. NcRNAs consist of around 60% of the transcriptional output of human cells. Large-scale transcriptome studies of mouse genomes also showed that ncRNAs are commonly transcribed. ncRNAs are generally classified via their functional and structural similarities: structural ncRNAs and regulatory ncRNAs. Structural ncRNAs have well-established structural functions, including rRNAs, tRNAs, spliceosomal small nuclear RNAs (snRNAs), and snoRNAs. Regulatory ncRNAs consist of miRNAs, interfering RNA, piwi-RNAs, long ncRNAs, and long intergenic ncRNAs, and they play roles in regulating gene expression. In this review, we mainly focus on the regulatory ncRNAs and also touch on several structural ncRNAs that are involved in skeletal development and diseases.

#### miRNAs

MiRNAs are highly conserved small ncRNAs with an average of 22 nucleotides in length. The first miRNA, lin4, was reported by Ruvkun and Ambros’ labs in 1993. Lin-4 was initially characterized as a gene that regulates the temporary development of C. elegans larvae in the 1980s [13, 14]. However, after more than ten years of research, it was demonstrated by Ruvkun and Ambros that instead of a protein-coding gene, lin-4 is a small ncRNA that contains a complementary sequence to that of the 3’untranslated region (3’UTR) of *lin-14* gene. Furthermore, the lin-14 protein was down-regulated at the post-transcriptional level through its 3’UTR [4, 15], indicating that lin-4 RNA regulates the expression of lin-14 protein at the post-transcriptional level. Since this revolutionary discovery, significant progress has been made in searching for miRNAs in different species and predicting and identifying their protein targets and functions.

In mammals, about half of all currently identified miRNAs are intergenic, which are regulated by their own promoters and transcribed independently. The remaining miRNAs are intragenic and mostly located in the introns of their host protein-coding genes. Most miRNAs are transcribed by RNA polymerase II [16] from DNA sequences into primary miRNAs (pri-miRNAs) transcripts. In the nucleus, pri-miRNAs undergo a processing step by the microprocessor complex, which includes RNA binding proteins (RBP), such as Drosha and DGCR8 [16–18]. This processing generates a hairpin precursor miRNAs (pre-miRNAs) with a length of ~ 70 nucleotides. Then, the pre-miRNAs are exported into the cytoplasm [19, 20], where they are cleaved by the RNase III enzyme Dicer, producing a miRNA duplex of approximately 22 nucleotides [21]. Finally, one strand of miRNA duplex is loaded into Argonaute proteins (AGO) to form the RNA-induced silencing complex (RISC) [22, 23]. The latest release (Version 22.1) of the miRbase, which is one of the most authoritative online resources for miRNA sequences and annotation established in 2002, contains around 48,000 mature miRNA sequences from more than 200 organisms, and in which about 2600 mature miRNAs from ~ 1900 hairpin precursors were identified in the human genome [24, 25].

Most miRNAs regulate gene expression by interacting with 3’UTR of target mRNAs to suppress protein translation and induce mRNA degradation [26, 27]. miRNAs can also mediate cell-cell communications by being secreted out of cells and transported to target cells by vesicles, including exosomes [28]. Abnormal expression of miRNAs is involved in various biological processes and pathological conditions, and miRNAs are crucial for animal development. For example, miRNA-mediated gene regulations play an essential role during skeletal development, bone and cartilage homeostasis, and the initiation and progress of skeletal disorders.

#### LncRNAs

LncRNAs are RNAs longer than 200 nucleotides that are not translated into proteins. They are transcribed by RNA polymerase I (Pol I), Pol II, and Pol III, and some of them are from processed introns [29]. The number of lncRNA is numerous. More than 100,000 lncRNAs have been found in the human genome [30, 31]. They are widely expressed and have specific expression patterns in physiological and pathological conditions. The biogenesis of most lncRNAs is similar to mRNAs. First, they are transcribed by polymerase II (Pol II), then undergo capping, polyadenylating, and splicing, producing transcripts similar to mRNAs. However, they are less abundant and conserved than the mRNAs [32]. Moreover, different from the cytoplasmic localization of mRNAs, a large proportion of lncRNAs are localized in the nucleus, which might be mediated by their less efficient processing rate [33, 34], nuclear retention element [35], and being tethered to chromatin [36]. A large fraction of lncRNAs is exported to the cytosol like mRNAs. Upon arrival in the cytoplasm, they are sorted into different organelles, such as mitochondria, or distributed in the cytoplasm. It is unclear how the ribosome binding property of cytoplasmic lncRNAs contributes to their functions.

The structure of lncRNAs is very flexible, making them capable of interacting with DNA, RNAs, and proteins [6]. Through these interactions, lncRNAs control gene expression at multiple levels. A large number of lncRNAs localize on chromatin, where they can interact with DNA, RNAs, or proteins, activating or silencing the transcription of the targeted chromatin [37–39]. One of the most prominent examples of chromatin localization lncRNAs is lncRNA XIST, which silences the gene expression of the whole X chromosome [40]. LncRNAs also regulate gene expression at the post-transcriptional and post-translational levels. They control the splicing, turnover, and translation of the mRNAs [41–43]. By directly binding to target proteins, lncRNAs modulate their subcellular localization, stability, and modification [44–46]. Benefitting from the long and flexible structure, several abundant lncRNAs function as scaffolds and assemble membrane-less organelles, e.g., lncRNA NEAT1 underlies the complex organization of paraspeckles [47], lncRNA HSAIII mediates the formation of nuclear stress bodies [47]. By virtue of these robust regulation mechanisms, lncRNAs regulate almost all biological processes, such as cell differentiation, proliferation, apoptosis, senescence, translation, and metabolism [6, 29]. LncRNAs often have a modular structure and are rich in repeats, which are increasingly shown to be relevant to their function [29]. The dysregulation of lncRNAs could lead to severe diseases, such as neuronal disorders, cancers, and bone diseases [48, 49].

#### CircRNAs

CircRNAs are a large class of widespread covalently closed single-stranded RNAs with tissue-specific and cell-specific expression patterns [50, 51]. More than 420,000 circRNAs have been identified in the human genome [52]. They are mainly produced from the back-splicing of the exons of mRNA precursors, with lengths varying from 100 nt to more than 4kb [53]. The intronic complementary sequences (ICSs) in introns flanking the circle-forming exons promote back-splicing by forming the transient pairs [54–56], which is mediated by the binding of RNA-binding proteins (RBPs) [57, 58]. Notably, most ICSs are Alu elements in humans. A small subset of circRNAs is also generated from the debranching failure of the intron lariats [59, 60]. Compared to linear RNAs, circRNAs prefer to localize in the cytoplasm [61–63], have lower expression levels [63–65], and are more stable [66].

circRNAs can also be divided into coding and non-coding circRNAs, like linear RNAs. Similar to lncRNAs, non-coding circRNAs exert their functions by interacting with DNA, RNA, and proteins. They can regulate gene transcription by directly binding to DNA. For example, CircRNA Ci-ankrd52 competes with the linear RNA cognates to form an R-loop with the second intron of the ANKRD52 locus. It promotes the transcriptional elongation of the linear RNA cognates [67]. Non-coding circRNAs can act as miRNA or protein sponges to regulate gene expression or signaling transduction. circRNA CDR1as contains 73 conserved miR-7 binding sites. It inhibits miR-7 by serving as a miR-7 binding platform [63, 68]. circRNA cia-cGAS is a circRNA antagonist for cGAS. It shows a more substantial binding capacity to cGAS than self-DNA. Thus, it inhibits the activation of cGAS by preventing it from binding to self-DNA [69]. Non-coding circRNAs can also form functional circRNP complexes to modulate signaling pathways, e.g., mitochondrial abundant circRNA SCAR forms an RNP complex with ATP5B, a regulator of the mitochondrial permeability transition pore (mPTP) complex. CAR-ATP5B RNP blocks the mPTP opening and inhibits the output of the mitochondrial reactive oxygen species (mROS) [70]. circVAMP3 promotes the formation of stress granules (SGs) by interacting with G3BP1 and CAPRIN1 [71].

Coding circRNAs are similar to linear mRNAs that function by generating proteins. Since the lack of a 5’ cap and 3’ poly (A) tail, circRNAs can only use the cap-independent translation elements, such as the embedded internal ribosome entry sites (IRESs), to initiate their translation [72–74]. N^6^-methyladenosine (m^6^A) modification can also drive the translation initiation of circRNAs [75]. Polysome profiling combined with mass spectrometry suggests that hundreds of circRNAs undergo m6A-driven translation [75, 76]. The cap-independent translation in normal conditions is inefficient, resulting in a low abundance of proteins translated from the circRNAs [72, 77]. Intriguingly, stress conditions significantly promote cap-independent translation efficiency [72, 75, 77], highlighting the regulatory roles of coding circRNA-derived proteins.

The stable advantage of research on circRNAs encourages people to develop RNA circle-based therapy technologies. RNA circles are engineered to aptamers [78, 79], antisense RNAs [80], miRNA and protein sponges [81–84], and protein synthesized templates [85], which show superior in their activity to their linear counterparts, to fulfill therapeutic purposes. In summary, circRNAs are essential regulators of physiological and pathological processes, and the accumulating knowledge of circRNAs helps identify novel circRNA therapeutic targets and biomarkers and develop novel therapeutic strategies.

#### Other ncRNAs

There is another group of ncRNAs that are around 70 to 200 nucleotides (nts) long and are transcribed by RNA polymerase III (Pol III) [86]. Examples of these classic ncRNAs include transfer RNAs (tRNAs), 5 S ribosomal RNA (5S rRNA), and U6 small nuclear RNA (U6 snRNA). These ncRNAs play crucial roles in cellular metabolism, and their varying expression is hard to understand. As a result, it has been generally considered that Pol III-transcribed genes are expressed constantly. Mature tRNAs have high and mostly constant expression levels due to extensive nucleotide modifications and secondary structure. Similarly, 5 S rRNA and U6 snRNA are abundant and expressed almost constantly. The Pol III transcription products consist of short, highly structured ncRNA and plays a crucial role in various cellular activities such as nuclear gene regulation, splicing, RNA maturation and stability, cytoplasmic protein targeting, and translation [87]. In higher eukaryotes, the Pol III transcriptome has expanded to include newly evolved ncRNA species that regulate autophagy, immune signaling cascades, and translation [88]. Dysregulation of Pol III transcription and dysfunction of ncRNA species are frequently observed in human diseases, such as severe viral infection outcomes, autoimmunity, and tumor progression.

#### Complex lncRNA/circRNA-miRNA post-transcriptional regulatory network

**Fig. 1:**
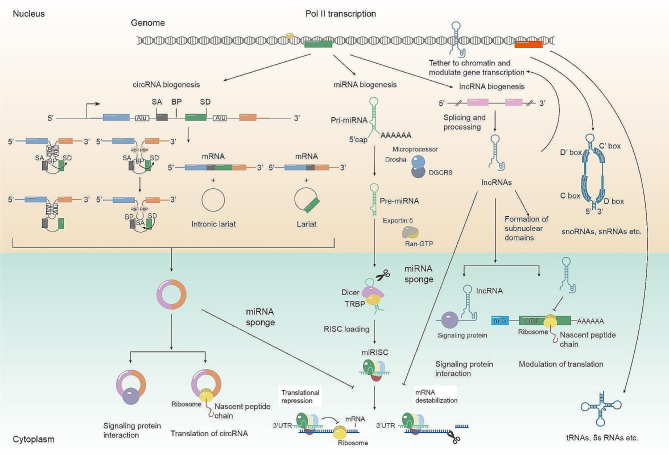
Biogenesis and functions of circRNAs, miRNAs, and lncRNAs. The precursors of most circRNAs, miRNAs, and lncRNAs are transcribed by polymerase II (Pol II) in a manner similar to that of linear mRNAs. During transcription, most human genes undergo competition between linear splicing and backsplicing of exons. Backsplicing is often favored due to factors, such as long flanking introns, inverted repeat elements (such as Alu), and RBPs [53]. The close proximity of a downstream splice-donor site (SD) with an upstream splice-acceptor site (SA) is achieved through the base pairing of inverted repeat elements or the dimerization of RBPs, leading to backsplicing. This process involves an upstream branch point (BP) attacking a downstream SD site, which in turn attacks an upstream SA site, ultimately resulting in the formation of exon-intron circRNAs or exonic circRNAs. CircRNAs have been shown to modulate cellular signaling transduction by interacting with signaling proteins, inhibiting miRNA functions, and serving as a template for the production of functional proteins. Within the nucleus, primary miRNA transcripts (pri-mRNAs) undergo cleavage mediated by the microprocessor complex Drosha–DGCR8, generating precursor miRNAs (pre-miRNAs) [16]. These pre-miRNAs are subsequently transported to the cytoplasm by exportin 5 [19, 20]. Once in the cytoplasm, pre-miRNAs undergo further cleavage by the enzyme Dicer, resulting in the formation of miRNA duplexes. These duplexes then associate with AGO proteins, thereby assembling the RNA-induced silencing complex (RISC). Once loaded onto RISC, miRNAs may undergo further processing, ultimately leading to their engagement with target messenger RNA (mRNA). Such interactions may result in mRNA destabilization or translational repression, representing key steps in post-transcriptional gene regulation. LncRNAs are transcribed and processed like mRNAs. However, many lncRNAs are retained in the nucleus to regulate the mRNA metabolism or form subnuclear domains. In the cytoplasm, lncRNA also modulates cellular signaling transduction by interacting with signaling proteins, inhibiting miRNA functions, and serving as a template for the production of functional peptides [6]

LncRNAs, circRNAs, and miRNAs are the most critical regulatory ncRNAs in cells. The binding of lncRNAs and circRNAs by miRNAs connects these three regulatory pathways and forms a post-transcriptional regulatory network. LncRNA/circRNA-miRNA regulation network seems to be unidirectional, in which lncRNAs and circRNAs inhibit miRNA activity, delivering the activation signals to mRNAs. However, RNA-RNA interactome studies identified tens of thousands of miRNA-lncRNA and miRNA-circRNA interaction pairs [89]. In these interaction pairs, one miRNA can target multiple lncRNA/circRNA targets, reminiscent of multiple mRNA targets of miRNAs. The RNA-RNA interactome study offers the possibility that miRNAs can regulate the function of lncRNAs/circRNAs, which allows the transfer of genetic information flow from miRNAs to lncRNAs/circRNAs. In addition, the binding of miRNAs in coding circRNAs may also inhibit their translation, which is a future direction worth exploring. Therefore, the lncRNA/circRNA-miRNA may form a complex regulatory network whose regulatory role is emerging (Fig. 1).

### ncRNAs in skeletal development

Small ncRNAs are essential for regulating the embryonic development of vertebrates. Deleting the critical miRNA-biogenesis enzyme Dicer in zebrafish and mice results in a lack of accumulation of miRNAs, leading to early embryonic lethality [90, 91]. Removal of Dicer in the limb bud mesenchymal cells by Prx1-Cre caused the developmental delay of limbs without obviously affecting their shape, suggesting Dicer is required for morphogenesis but not patterning of the vertebrate limbs [92].

#### Regulation of ncRNAs in endochondral ossification

Endochondral ossification is a developmental process that forms long bones in skeletal systems [93]. It begins with the condensations of mesenchymal stem cells, which differentiate into chondrocytes expressing transcription factor Sox9, extracellular matrix protein Col2a1, and polyproteoglycans (Aggrecan) to form cartilage templates. Chondrocytes continue to expand the cartilage template through continuous proliferation. Then, the cells in the middle of the cartilage template stop proliferating and differentiate into hypertrophic chondrocytes, expressing the extracellular matrix type X collagen encoded by the *Col10a1* gene. As blood vessels begin to invade the cartilage template and are accompanied by the calcification of hypertrophic chondrocytes, hypertrophic chondrocytes are subsequently replaced by osteogenesis and form a primary ossification center. The growth plate is a temporary cartilage tissue located near both ends of the long bone between the primary and secondary ossification center. The proliferation and differentiation of growth plate chondrocytes contribute to the lengthening of the long bones.

Dicer-related signaling pathway plays an important role in the regulation of skeletal development and growth in mice. Conditionally deleting Dicer in mouse growth plates led to the lethality around weaning time with skeletal growth defects caused by a reduction of proliferation and accelerated terminal differentiation of chondrocytes [94]. Specifical deletion of Dicer in mature osteoblasts by the Osteocalcin-Cre increased bone mass in adult mice from 2 months to 8 months in long bones and vertebrae [95].

**Fig. 2:**
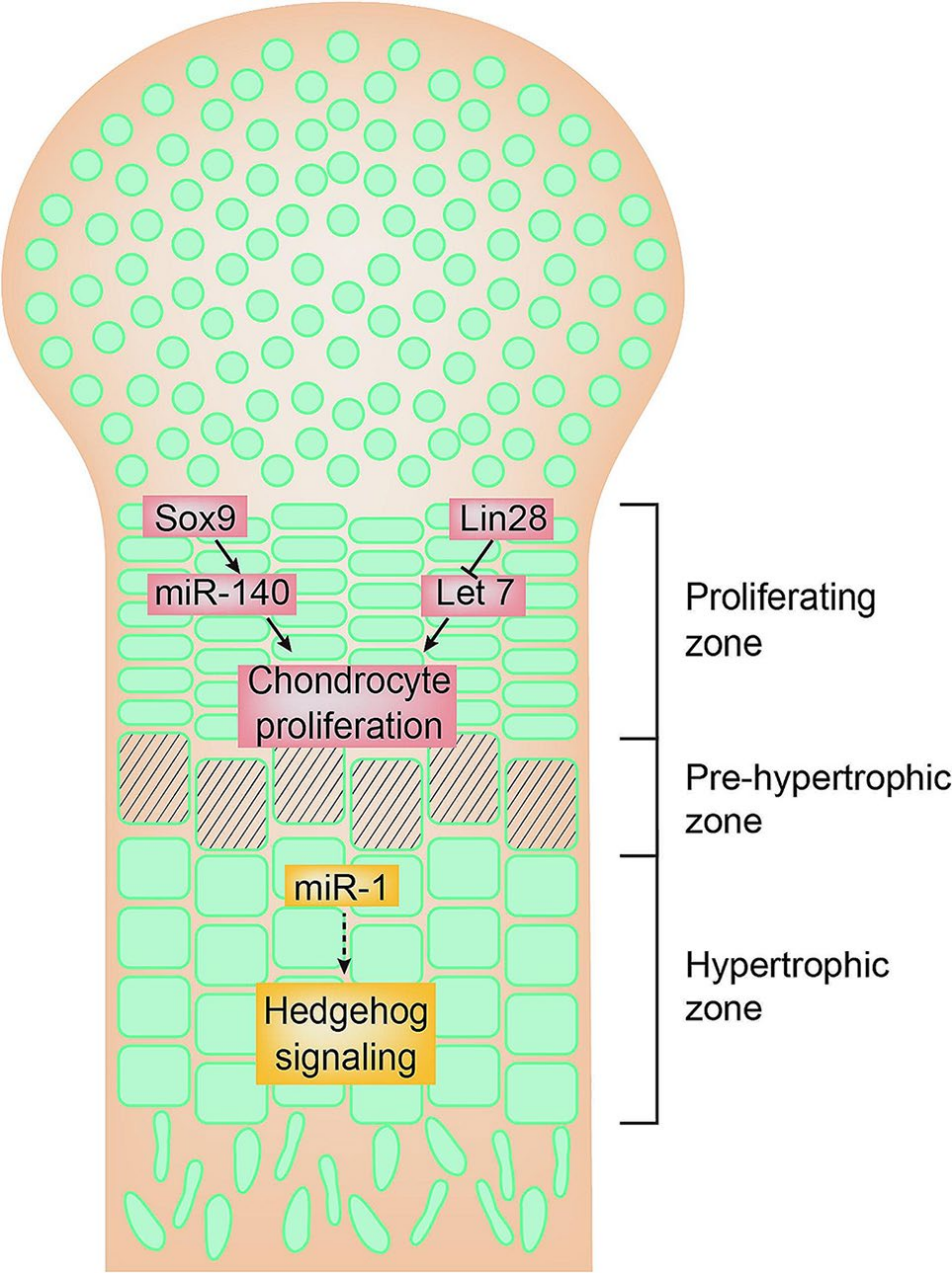
miRNAs regulating growth plate development. MiR-140 is expressed in the proliferating chondrocytes regulated by transcription factor Sox9. MiR-140 coordinately functions with let7, which is regulated by Lin28, to regulate chondrocyte proliferation in the growth plate. miR-1 is mainly expressed in the hypertrophic chondrocytes of growth plates in mice. miR-1 may regulate hedgehog signaling in hypertrophic chondrocytes

Among hundreds of miRNAs, miR-140 is one of the most extensively studied miRNAs that are closely associated with cartilage development. miR-140 is believed to be expressed explicitly in cartilage but not in other tissues based on whole-mount in situ hybridization data in zebrafish and mouse embryos [96, 97]. MiR-140 is highly expressed in proliferating chondrocytes and some pre-hypertrophic chondrocytes but not in hypertrophic chondrocytes in post-natal growth plates in mice (Fig. 2). Global knockout of miR-140 in mice caused shortened body length and tail length with a slightly shortened proliferation zone and proportionally expanded resting zone of the growth plate [98]. Interestingly, mice with both miR-140 deficiency and suppression of let 7 by overexpression of Lin28a led to dramatic skeletal growth defects. In contrast, overexpression of Lin28 only results in a mild abnormality of cartilage and bone in mice, suggesting lin-7 and miR-140 can work coordinately to regulate cartilage development in vivo [99]. MiR-140 was reported to regulate palatogenesis by targeting 3’UTR of Pdgf-receptor alpha gene and down-regulating Pdgfa protein to inhibit the accumulation of cranial neural crest cells to the oral ectoderm, and its deficiency caused an altered shape of the palate in zebrafish [100]. A single nucleotide mutation in the seed region of pre-miR-140 was reported to generate a gain of functions of miR-140-5p and to be associated with autosomal dominant human skeletal dysplasia characterized by disproportionate short stature with smaller limbs, which is the first reported human disease caused by the gain-of-function mutations in a highly conserved miRNA [101]. Genetic evidence in zebrafish shows that Sox9 is upstream of miR-140 to regulate chondrogenesis [102]. However, the direct target genes and signaling pathways regulated by miR-140 controlling the development of growth plates remain to be determined in vivo.

miR-1 is detected by in situ hybridization to be highly expressed in the hypertrophic chondrocytes compared to that in proliferating chondrocytes in pre-natal chicken growth plates [103]. Transgenic mice overexpressing miR-1 in resting and proliferating chondrocytes in postnatal growth plates resulted in smaller stature with shortened limbs by decreasing the proliferation rate of growth plate chondrocytes, disrupting their hypertrophic differentiation and inhibiting the apoptosis of chondrocytes in lower hypertrophic zone [104]. Hedgehog signaling is significantly decreased in the pre-hypertrophic chondrocytes of growth plates in the miR1-overexpressing mice. However, the direct target evidence has not been reported in vivo.

**Fig. 3:**
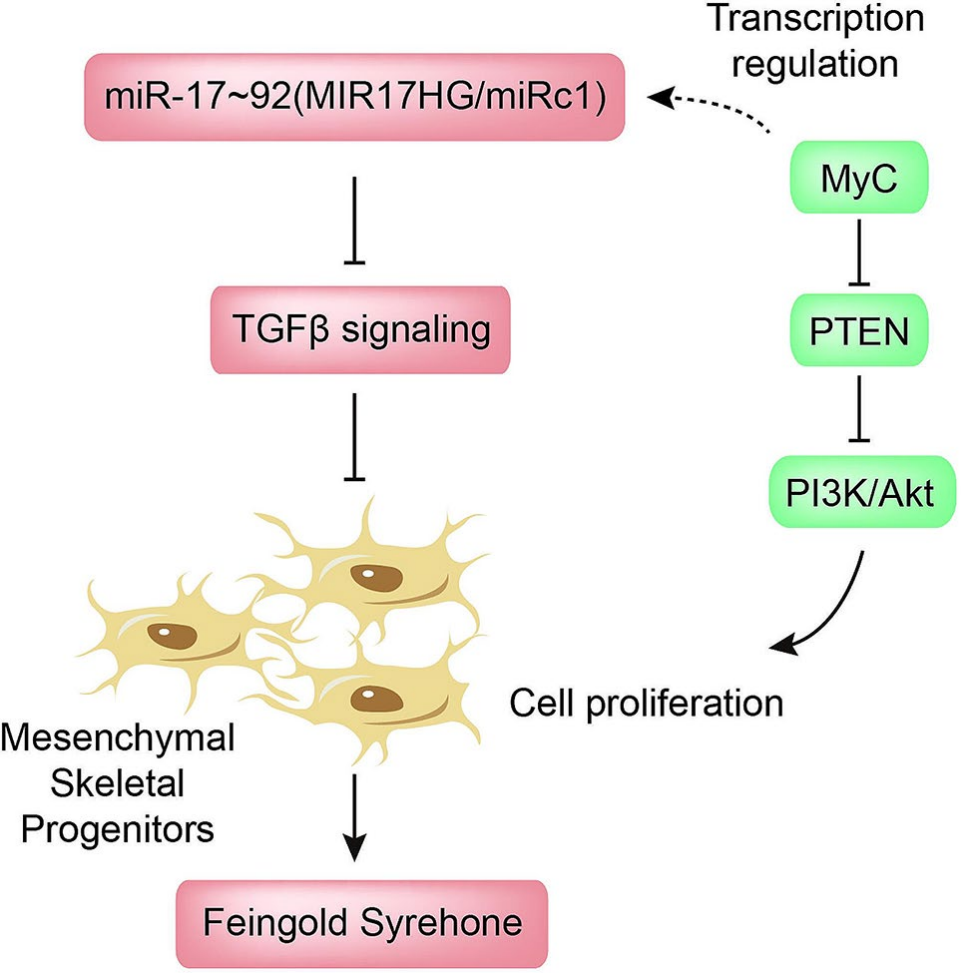
miRNAs controlling the proliferation of mesenchymal stem cells. Mutations of the miR-17 ~ 92 cluster are associated with human Feingold Syndrome. miR-17 ~ 92 inhibited TGFβ signaling, thus suppressing the proliferation of skeletal mesenchymal progenitors

MiR-17 ~ 92 cluster is a classic polycistronic miRNA encoded by the *MIR17HG* gene (also referred to as *Mirc1* gene) containing miR-17, miR-20a, miR-18a, miR-19a, miR-19b-1, and miR-92a-1. Haploinsufficiency of miR-17 ~ 92 leads to a human autosomal dominant syndrome, referred to as Feingold syndrome, characterized by microcephaly, short stature, and severe digital abnormalities (Fig. 3). Homozygous deletion of the miR-17 ~ 92 cluster leads to prenatal lethality in mice with a severe delay of bone formation mimicking the defects in Feingold syndrome. Transgenic mice lacking one allele of the miR-17 ~ 92 cluster were viable but with significantly smaller stature than wild-type littermates [105, 106]. Different miRNAs in this cluster were demonstrated to functionally cooperate to control the patterning and development of the skeleton in vertebrates.

MiR-322 is critical for the development and growth of tracheal cartilage. Lacking miR-322 in chondrocytes results in the upregulation of MEK1 protein, thus inhibiting the growth of tracheal cartilage, leading to respiration failure in mice [107]. Since lncRNA and circRNA have been identified only in recent years, there have been few reported mouse genetic studies on lncRNA or circRNA to show their roles in cartilage development in vivo. miR-34 was demonstrated to regulate bone formation by targeting SATB2 and inhibiting the proliferation and differentiation of osteoblasts in mice [108].

#### Regulation of ncRNAs in intramembrane ossification

Several miRNAs associated with intramembrane bone formation in vivo have been reported. Conditional deletion of Dicer in Osx-positive cells in mice resulted in a delay in suture closure time and a disorder of calvarial morphology in the skull development [109]. Specifically, deleting Dicer in mature osteoblasts by Osteocalcin-Cre did not change bone mass in the calvariae bone. Heterozygous miR-17 ~ 92 in humans was demonstrated to lead to microcephaly, which is one of the typical phenotypes of Feingold syndrome [105]. Lacking miR-92a in zebrafish led to obvious defects in craniofacial skeleton development. MiR-92a is highly expressed in the chondrogenic progenitor in zebrafish and regulates pharyngeal cartilage formation by directly targeting the noggin3-modulated Bmp signaling pathway [110]. Nardocci et al. [111] used mouse preosteoblastic cells and induced them to differentiate into osteoblasts. They performed RNA sequencing and identified lncRNA candidates that showed differential expression patterns during osteoblast differentiation. LncRNA-1 was found to be upregulated during osteogenesis and downregulated during myogenesis. Knockdown of lncRNA-1 expression inhibited osteogenic differentiation in primary mouse preosteoblasts, suggesting a new regulatory RNA involved in the early stages of osteogenesis.

### ncRNAs in skeletal diseases

Epigenetic regulation is crucial for the appropriate development of the skeletal system. Emerging evidence indicates that ncRNAs are critical epigenetic factors that participate in bone development, homeostasis, and repair processes. Many downstream genes may be interfered with by ncRNAs and thereby alter cellular pathways and networks. Hence, changes in levels or activity of ncRNAs can impact cellular processes involved in proper skeletal development, including proliferation, metabolism, apoptosis, and differentiation. As epigenetic modulators are critically involved in the control of gene expression, ncRNAs are involved in the pathogenesis of a variety of skeletal diseases, such as osteoarthritis, osteoporosis, intervertebral disc degeneration, and rheumatoid arthritis.

## ncRNAs in intervertebral disk degeneration

Intervertebral disc degeneration (IVDD) is a common degenerative musculoskeletal condition associated with many elements, including age, genetics, mechanics, and lifestyle [112]. Low back pain and reduced lumbar spine support are typical symptoms of IVDD, remarkably affecting the quality of life of patients and aggravating social burdens globally [113]. The intervertebral disc consists of the inner nucleus pulposus (NP) with the annulus fibrosus around it. Nucleus pulposus cells (NPCs) are the major type of cells residing in the NP, playing an essential role in maintaining disc health [114]. Excessive apoptosis of NPCs and degradation of their ECM result in IVDD.

**Fig. 4:**
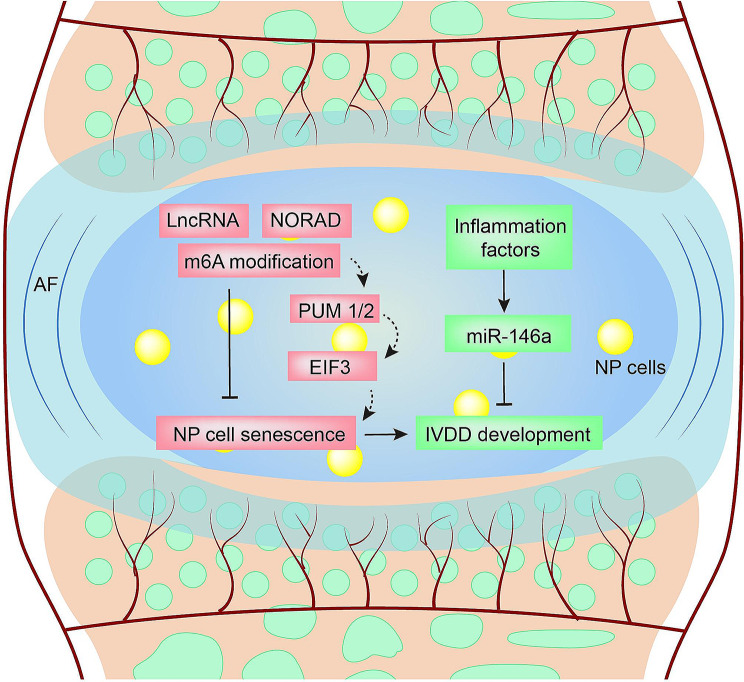
NcRNAs associated with IVDD. LncRNA NORAD regulates m6A modifications to regulate NP cells senescence. Inflammation factors are demonstrated to trigger the expression of miR-146a in NP cells to inhibit IVDD development

Emerging evidence demonstrates that non‑coding RNAs are linked to the pathogenesis of IVDD [115, 116]. A number of aging-associated diseases are accompanied by epigenetic alterations [117]. NORAD is a lncRNA that regulates DNA damage and maintains genome integrity during cell growth and aging [83]. Li et al. [118] found that NORAD was significantly reduced as the development of NPC senescence increased, while elevating it alleviated NPC senescence (Fig. 4). They constructed NORAD global knockout mice and found that KO mice manifested premature aging with pronounced kyphosis at the age of 10 weeks. Similarly, the radiographic and histological analyses indicated that IVDD occurred in NORAD^−/−^ mice at the age of 8 weeks, characterized by worse MRI manifestation, collapsed disc space, narrowed endplates height, and impaired cellularity of the NP. Furthermore, degeneration of NP and IVDD was more obvious in KO mice than in WT mice in an IVDD mouse model, suggesting a pivotal role of NORAD in the pathogenesis of IVDD. Mechanistically, NORAD was involved in m6A modification owing to increased WTAP expression, which was correlated to the trimethylation of H3K4. NORAD sequestered PUM1/2 and ameliorated the pro-decay of NPCs, and the role of NORAD was impaired in senescent NPCs by elevating m6A-mediated decay. In addition, PUM1/2 could target and degrade transcripts of E2F3, an essential factor of the cell cycle and cell proliferation, resulting in NPC senescence. Interruption of NORAD m6A modification could serve as an underlying therapeutic target of IVDD.

MiR-146a has been broadly investigated in the skeletal diseases [119–123]. Besides osteoarthritis and rheumatoid arthritis, it also seems to be correlated to the pathogenesis of IVDD. Gu et al. cultured disc segments from miR-146a^−/−^ mice and WT mice under IL-1 challenge ex vivo [124]. IL-1 induced proteoglycan, MMP-13, and ADAMTS-5 expression in the disc organ cultures both in the WT group and KO group, which are correlated to progressive degenerative diseases, such as OA and IVDD. However, depletion of miR-146a resulted in more profound effects, indicating that stimulation of miR-146a might protect from IVDD and inflammation. In vivo experiments are required to verify the above results.

Numerous studies have demonstrated that ncRNAs have critical functions in modulating IVD development (as discussed in recently published review articles [115, 125]). Besides, some ncRNAs, including miR-15a-5p [126], miR-19b-3p [127], miR-25 [128], miR-26a-5p [129], miR-105-5p [130], miR-130b [131], miR-145-5p [132], miR-195 [133], miR-345-3p [134], miR-495-3p [135], miR-499a-5p [136], miR-502 [137], miR-660 [138], miR-2355-5p [139], miR-4478 [140], circKIF18A [141], hsa_circ_0083756 [142], hsa_circ_0059955 [143], circSPG21 [144], circPKNOX1 [145], circ-FAM169A [146], circERCC2 [147], circ-4099 [148], lncRNA XIST [149], and LncRNA NR2F1-AS1 [132] are revealed to be differently expressed in NP tissues and cells from IVDD patients. However, the investigation into the different mechanisms of IVDD in genetic animal models is extremely limited. Therefore, exploring the diverse mechanisms participating in NPC senescence is crucial for investigating IVDD, which will facilitate the development of novel therapeutic strategies.

## ncRNAs in rheumatoid arthritis

Rheumatoid arthritis (RA) is a systemic inflammatory autoimmune disease without a clear etiology. RA is characterized by chronic, destructive arthritis due to multiple changes in the adaptive immune system and innate immune system [150]. T cells, B cells, osteoclasts, and many cytokines are involved in the pathogenesis of RA [151]. In addition, ncRNAs have been shown to exert powerful regulatory functions in the immune response, including the regulation of lymphocytes and dendritic cells.

MiR-155 is a widely investigated miRNA in autoimmune diseases, including RA. In RA patients, the expression of miR-155 was dramatically elevated in the peripheral blood mononuclear cells compared with that in the healthy counterparts [152]. MiR-155 globally knockout mice showed defective B cell immunity and aberrant production of pro-inflammatory cytokines, which were strongly correlated to the pathogenesis of RA [153]. In a collagen-induced RA (CIA) model, miR-115^−/−^ mice developed much more moderate typical clinical and histological signs than WT mice [154]. This might be due to fewer anti-collagen antibodies and antigen-specific T cells in KO mice. In addition, the levels of interleukin-6 (IL-6), IL-17, and IL-22 were reduced, indicating that Th17 polarization of KO mice was hampered. Moreover, miR-155 could directly target human Src homology 2-containing inositol phosphatase-1 (SHIP-1) for degradation. Expression of SHIP-1, a potent antagonist of several inflammatory pathways, was markedly decreased in RA synovial CD14^+^ cells. On the contrary, suppression of miR-155 with antagomir dramatically elevated the expression level of SHIP-1 mRNA in synovial fluid CD14^+^ cells from RA patients. Chen et al. found that the bone marrow-derived dendritic cells (BMDCs) obtained from the KO mice generated fewer pro-inflammatory cytokines in the lipopolysaccharide (LPS) induced inflammation model [155]. Collectively, these findings suggest that miR-155 is critical for the pathogenesis of RA, and it might benefit the development of novel therapeutic strategies.

MiR-146a is a broadly investigated anti-inflammatory miRNA [156]. MiR-146a global knockout mice presented aggravated joint inflammation, while intravenous injection of miR-146a mimics prevented joint destruction in an RA model [119, 121]. Ammari et al. found that the expression of miR-146a was decreased in the Ly6C^high^ monocytes of arthritic mice and CD14^+^ CD16^−^ monocytes in RA patients. They administrated miR-146a mimics targeting Ly6C^high^ monocytes through DMAPAP/DOPE cationic liposome. The administration reduced bone absorption and bone erosion in arthritic joints in KO mice. Therefore, overexpression of miR-146a in Ly6C^high^ monocytes could alleviate joint destruction and avoid inflammation in RA simultaneously.

MiR-204 and miR-211, two homologous miRNAs with the same target genes, were reported to protect against OA progression [157]. Wang and coworkers continued to determine the effects of two miRNAs on synovial hyperplasia and inflammation [158]. First, they found that expression of miR204/211 was dramatically decreased in synoviocytes of CIA mice, affecting cell migration, apoptosis, proliferation, and inflammatory responses. Based on bioinformatics analysis and subsequent validation experiments, *Ssrp1* was considered to be a target gene of miR-204 and miR-211. Next, they constructed miR204/211 double knockout mice and performed the CIA model. Double knockout mice presented higher Ssrp1 levels with stronger synovial hyperplasia and inflammation, which could be restored by injecting AAV- shSsrp1 intra-articularly. These results indicate that miR204/211 is essential in the progression of RA, which might provide a novel treatment strategy for RA.

Nuclear-enriched abundant transcript 1 (NEAT1) is a lncRNA that was reported to play a vital role in the progression of RA [159, 160]. The synovial tissue obtained from both mice and RA patients expressed higher NEAT1 [159]. Depleting NEAT1 with lenti-sh-NEAT1 in mice inhibited the infiltration of both CD4^+^ T cells and macrophages into synovial tissue [159, 160]. Moreover, it reduced the expression of inflammatory factors in peripheral blood and decreased the incidence/severity of RA in CIA mice [159]. Mechanistically, NEAT1 was activated by p-p65 and then promoted the p300/CBP/IL-18 signaling pathway, which eventually aggravated RA [159]. NEAT1 could also stimulate CD4^+^ T cells to differentiate into Th17 cells by enhancing the STAT3 expression [160].

To date, nearly 100 ncRNAs have been reported to be strongly associated with the pathogenesis of RA (presented in a recently published review article [161]), which still needs to be studied and validated further by genetic models. Taken together, the pivotal roles of ncRNAs as biomarkers and targets for RA require additional research.

## ncRNAs in osteoporosis

Bone metabolism is a complicated process that includes anabolism and catabolism. Osteoblasts and osteoclasts play essential roles in maintaining the homeostasis of the formation and absorption of the bones. Once bone formation decreases and bone absorption increases, osteoporosis occurs and aggravates the fracture risk. Emerging evidence indicates that ncRNAs are closely involved in the incidence and development of osteoporosis. Jin et al. [162] found that more than 300 ncRNAs were aberrant in patients with postmenopausal osteoporosis, and regulating these related ncRNAs might be helpful in treating osteoporosis. In this section, we will review those pivotal ncRNAs whose roles have been verified through genetic animal models.

### Bone formation

#### Positive regulators

**Fig. 5:**
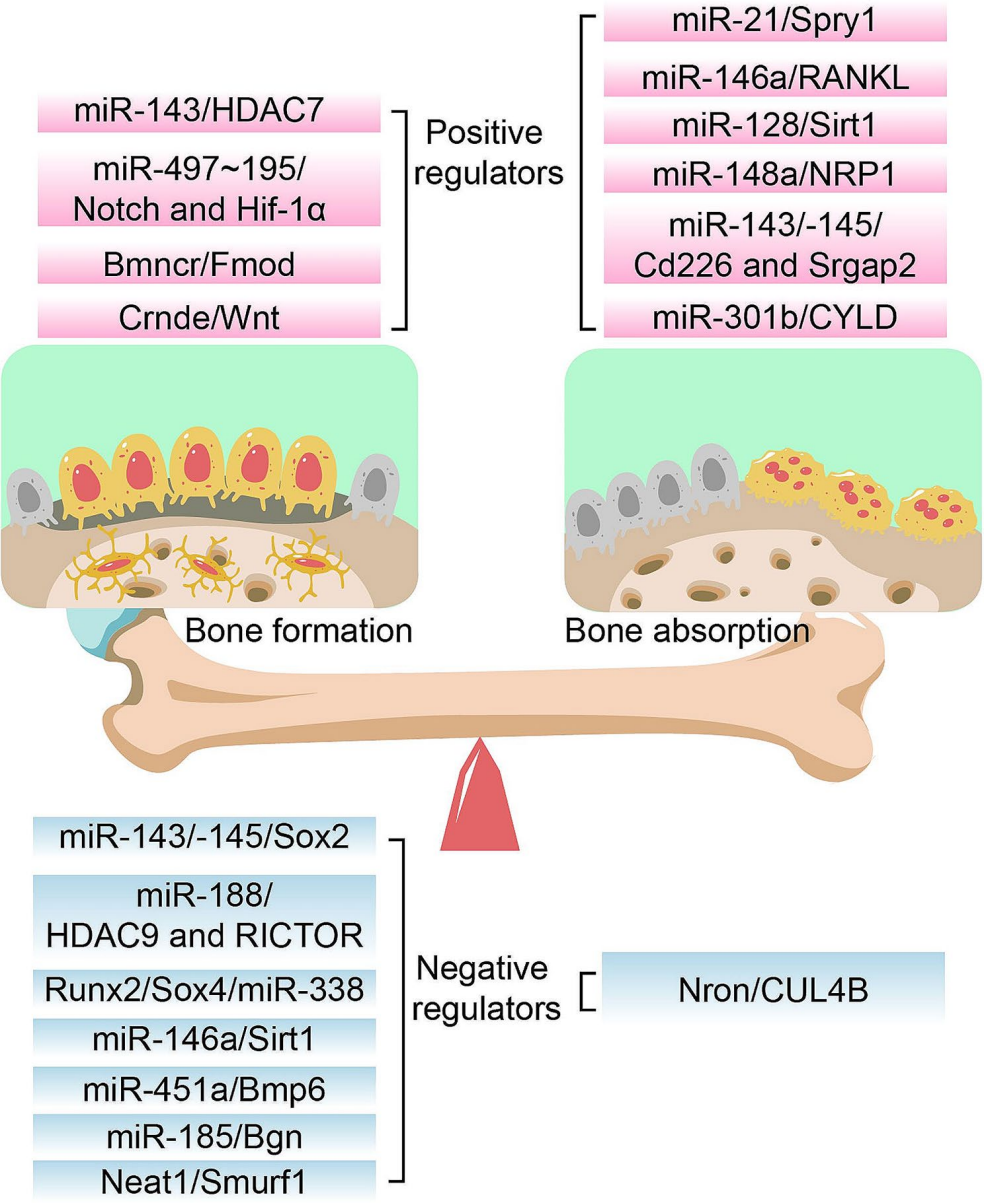
A diagram briefly summarizing the ncRNAs that are involved in the dynamic balance between osteogenesis and osteoclastogenesis in transgenic mice models. The pathological progress of OP is due to the imbalance between bone formation and resorption mediated by osteoblasts and osteoclasts, respectively. Dysregulated ncRNAs participate in the development of OP

Osteoblasts, mainly differentiated from mesenchymal stem/stromal cells, regulate bone homeostasis by facilitating bone formation. Some ncRNAs have been investigated to facilitate osteoblastic differentiation in osteoporosis. MiR-143 is highly expressed in osteoblasts, and overexpression of it was reported to promote osteoblast differentiation [163]. Osteoblastic differentiation of miR-143^−/−^ mice was markedly inhibited. Using mRNA-sequencing combined with target prediction and luciferase reporter experiment, miR-143 was found to directly target the 3’-UTR of HDAC7 and inhibit HDAC7 expression in osteoblasts. HDAC7-siRNA was injected into the bone marrow of miR-143 KO mice, and the symptoms caused by miR-143 depletion were significantly ameliorated. On the contrary, administration of agomiR-143 intravenously accelerated osteogenesis and inhibited bone loss in an aging-induced osteoporosis mice model. MiR-497 ~ 195 cluster tended to be expressed in CD31^hi^ Emcn^hi^ endothelial cells (a specific bone vessel subtype), which was decreased during aging [164]. The endothelial-specific knockout of miR-497 ~ 195 cluster using miR-497 ~ 195^fl/fl^; Cdh5 (PAC)-Cre mice manifested fewer CD31^hi^Emcn^hi^ vessels and lower bone mass while intravenous injection of aptamer-agomiR-195 promoted bone formation in aged mice. Mechanistically, the miR-497 ~ 195 cluster maintained the Notch activity via targeting F-box and WD-40 domain protein (Fbxw7) and maintained the HIF-1α stability via targeting Prolyl 4-hydroxylase possessing a transmembrane domain (P4HTM) in the endothelial cells. LncRNA Bmncr determined the fate of aging BMSCs by regulating the osteogenic niche in the bone marrow [165]. Bmncr^−/−^ mice presented redundant age-associated bone loss, while transgenic overexpression of Bmncr increased the bone mass. Bmncr served as a scaffold to promote the interaction between TAZ and ABL and then stimulated the assembly of the TAZ-RUNX2 and TAZ-PPARG transcriptional complex, resulting in bone formation. Another lncRNA, Crnde, also has a function of osteoblast proliferation and differentiation [166]. Crnde knockout mice possessed impaired osteoblast proliferation/differentiation and reduced bone mass phenotypes. This might be caused by decreased Alp, Runx2, and Osx expression via inhibiting the Wnt/β-catenin pathway, which was responsible for cell proliferation (Fig. 5).

#### Negative regulators

Some other ncRNAs have been investigated to be negative regulatory factors of osteogenesis. Due to osteoporosis being an aging-related disease, Li and coworkers performed miRNA microarray analysis to compare bone marrow stromal cells (BMSCs) in young mice with those in aged mice and found that miR-188 expression was much higher in aged mice [167]. Global knockout of miR-188 or administration of aptamer-antagomiR-188 into bone marrow alleviated age-related bone loss and promoted adipogenesis. However, overexpression of miR-188 in osterix^+^ osteoprogenitors or BMSCs resulted in aggravated bone loss and adipogenesis of BMSCs. Moreover, HDAC9 and RICTOR were validated as the direct targets of miR-188. MiR-146a^−/−^ mice have an enhanced osteoblastic activity and bone formation rate [122, 123]. MiR-146a-5p expression was increased in the bone tissue of both aging female mice and PMOP patients [122]. In addition, miR-146a^−/−^ reversed bone loss while aging in female mice. Meanwhile, the multiplication of MSC was faster, and apoptosis of MSC was weaker in miR-146a^−/−^ mice with higher expression of osteoblastic markers, including Alp and Ocn. Using target prediction, luciferase reporter experiment, and RNA immunoprecipitation assays in MC3T3-E1 cells, Zheng et al. [122] found Sirt1 could bind with miR-146a-5p in its 3’-UTR. Furthermore, the protein expression of Sirt1 was significantly elevated in miR-146a global KO mice. Thus, miR-146a was positively associated with age, and miR-146a ablation could prevent mice from OP. LncRNA Neat1 acted as a vital ncRNA in osteoblastic differentiation in mice subjected to mechanical loading [168]. Neat1^−/−^ mice showed impaired bone structure and bone loss. In addition, Neat1 depletion inhibited bone formation induced by mechanical loading and blunted mechanical unloading-induced bone loss. Mechanistically, paraspeckles accelerated the nuclear retention of Smurf1 suppressed the translation, and ultimately suppressed ubiquitination-mediated degradation of Runx2, one of the targets of Smurf1.

Postmenopausal osteoporosis (PMOP) is a frequent bone disorder. Xu et al. [169] found miR-143 and miR-145 were potential treatment targets for PMOP, and overexpressing miR-143/145 hampered self-renewing and osteoblastic differentiation of BMSCs. On the contrary, miR-143/145 global knockout or antagomiR-143/145 significantly ameliorated estrogen-deficient OP in female mice. Specifically, cytoplasmic miR-143/145 and lncRNA MIR143HG, controlled by ERβ, cooperatively regulated pluripotency genes translation via canonical ceRNA pathway, and MIR143HG cooperated with miR-143 to nuclear translocation for co-activation of SOX2 transcription via opening promoter chromatin. In an ovariectomized (OVX) mouse model, miR-185 knockout ameliorated bone loss in an estrogen-deficient OP model, while miR-185 agomir reversed the increased bone formation in miR-185^−/−^ mice after OVX [170]. Osteoblasts and MSCs obtained from miR-185^−/−^ mice displayed enhanced bone formation. Mechanistically, miR-185 ablation activated the BMP/Smad pathway to regulate Bgn, the knockdown of which reversed the increase of Alp, Bmp2, and p-Smad1/5/8. MiR-338 cluster (including miR-338-3p and miR-3065-5p) was highly enriched in PMOP patients and OVX-induced mice [171]. Either miR-338-3p or miR-3065-5p inhibitor could dramatically alleviate the symptoms of OP in OVX mice by suppressing the expression and secretion of the miR-338 cluster. Moreover, the deletion of miR-338 could relieve or even arrest OP progression. BMSCs obtained from the femurs of miR-338 global knockout mice expressed higher Runx2, Opn, Osx, and Ocn with a greater number of mineralization nodules after osteogenic induction. Dual luciferase assay results validated that overexpression of miR-338-3p and miR-3065-5p in MC3T3-E1 cells inhibited osteoblastic differentiation by directly targeting Runx2 and Sox4. Lu et al. [172] found that miR-451a was dramatically elevated post-OVX operation. Primary osteoblasts extracted from miR-451a^−/−^ mice possessed the stronger proliferative ability, Alp activity, and the ability to form a mineralized matrix. The gene and protein levels of osteoblast markers were dramatically increased in osteoblasts harvested from miR-451a^−/−^ mice. Additionally, ablation of miR-451a alleviated the bone loss in estrogen-deficient OP, which was abrogated by miR-451a agomir administration. Using database prediction and further validation of biological experiments, Bmp6 was considered a direct target of miR-451a. Table 1 summarizes the role of some ncRNAs in osteoporosis and their mechanisms through bone formation validated in the transgenic mice model.

**Table 1:** The role of some ncRNAs in osteoporosis and their mechanisms through bone formation validated in the transgenic mice model

ncRNA type	ncRNA name	Target genes and pathways	Transgenic mice	Function	Ref
miRNA	miR-143	HDAC7	miR-143-/-	Promoting angiogenesis coupling with osteoblast differentiation	[163]
miRNA	miR-143/145	Sox2	miR-143/145-/-	miR-143/145 overexpression impaired BMSCs self-renewing and osteoblastic differentiation function	[169]
miRNA	miR-146a	n.a	MiR-146a-/-	miR-146a inhibited the proliferation and osteoblast differentiation but accelerated apoptosis of MSC	[123]
miRNA	miR-146a-5p	Sirt1	miR-146a-/-	miR-146a-5p inhibited the osteoblast differentiation of BMSCs; miR-146a-5p deletion protected female mice from age-induced bone loss	[122]
miRNA	miR-185	Bgn/BMP/Smad signaling	miR-185-/-	Redundant bone formation after miR-185 depletion	[170]
miRNA	miR-188	HDAC9 and RICTOR	miR-188–/–, osterix + miR-188-Tg mice	Inhibition of miR-188 increased bone formation and decreased bone marrow fat accumulation in aged mice	[167]
miRNA	miR-338	Runx2/Sox4/miR-338 signaling	miR-338-/-	Deletion of the miR-338 cluster or injection of a miR-338 cluster inhibitor prevented osteoporosis after ovariectomy	[171]
miRNA	miR-451a	Bmp6/SMAD1/5/8	miR-451a-/-	Osteoblasts and MSCs isolated from miR-451a KO mice showed promoted osteogenesis	[172]
miRNA	miR-497 ~ 195 cluster	Notch and HIF-1a	miR-497 ~ 195 fl/fl; Cdh5 (PAC)-Cre	Promoting angiogenesis coupled with osteogenesis; target for age-related osteoporosis	[164]
LncRNA	Bmncr	FMOD; TAZ RUNX2/PPARG interation	Bmncr-/-	Restoring BMNCR levels in human BMSCs reversed the age-related switch between osteoblast and adipocyte differentiation	[165]
LncRNA	Crnde	Wnt/β-catenin signaling	Crnde-/-	Crnde knockout impaired osteoblast proliferation and differentiation	[166]
LncRNA	Neat1	Smurf1/Runx2	Neat1-/-	Neat1 deficiency in osteoblasts reduced the response of osteoblasts to mechanical stimulation	[168]

### Bone resorption

Osteoclasts are differentiated from macrophage/monocyte precursor cells in the presence of RANKL and other osteoclastogenic factors. Understanding aberrant bone resorption is critical for investigating the diagnosis, treatment, and prevention of osteoporosis. Mir-21 is the first investigated ncRNA in regulating bone resorption in mice. miR-21^−/−^ mice showed normal skeletal structure and osteogenesis while inhibited osteoclastogenesis [173]. However, miR-21^−/−^ mice exhibited inhibited bone resorption in tartrate-resistant acid phosphatase (TRAP) staining and C-telopeptide of type 1 collagen (CTX-1) ELISA test. Mir-21 ablation also attenuated OVX-induced osteoporosis and aging-induced osteoporosis, which was validated in osteoporosis patients. PDCD4, a regulator of osteoclast differentiation, was proved to be a direct target of miR-21 in supporting osteoclastic function. Mir-128 was regarded as an essential ncRNA in aging and inflammatory-related diseases involving osteoporosis [174]. MiR-128 was highly enriched in PMOP patients and OVX mice, which was further proved to promote osteoclastogenesis. Depletion of miR-128 in the osteoclasts (using *miR-128*^*flox/flox*^; *LysM-Cre* mice) dramatically decreased osteoclastogenesis and relieved OVX-induced bone loss, as evidenced by TRAP staining, qPCR analysis, and ELISA assay. MiR-128 promoted osteoclastogenesis by targeting Sirt1 and then activating NF-κB signaling by acetylation modifications. Xu et al. [169] demonstrated that miR-143/145 was notably increased in the serum, saliva, and BMSCs from postmenopausal women compared to premenopausal counterparts. In addition, the self-renewing and differentiation of BMSCs were impaired in postmenopausal women. Depletion of miR-143/145 or injection of antagomiR-143/145 arrested bone loss and retained bone regeneration in the estrogen-deficient OP, while agomiR-143/145 exacerbated it. That was due to extracellular vesicles from BMSCs loading miR-143/145 and being transferred to osteoclasts to affect osteoclastic activity and function by targeting Cd226 and Srgap2. Besides miR-143/145, miR-146a, miR-148a, and miR-301-b were also regarded as therapeutic targets for estrogen deficiency-induced osteoporosis. MiR-146a was elevated in OVX-induced OP mice, the deletion of which protected the mice from bone loss [123]. Osteoclast activities were hampered in the miR-146a global knockout mice subjected to OVX. Macrophage colony-stimulating factor (M-CSF) and RANKL/OPG ratio from the bone microenvironment regulated osteoclastogenesis under estrogen deficiency. Pan et al. revealed that miR-148a^−/−^ mice showed an increased bone mass and decreased bone resorption by targeting NRP1 [175]. In an estrogen-deficient OP model and a calvarial osteolysis model, depletion of miR-148a prevented mice from redundant bone absorption, while injection of agomiR-148a intravenously or AAV-shNRP1 via bone marrow cavity accelerated pathogenesis of OP. More importantly, β-CTX secretion in PMOP patients was positively related to the expression of miR-148a. MiR-301-b was elevated in the bone tissue of either PMOP patients or OVX-induced mice [176]. Osteoclastic conditional knockout of miR-301 (*miR-301-b*^*flox/flox*^; *LysM-Cre* mice) led to reduced osteoclastogenesis and osteoclast number, exerting a strong bone protection effect. Further studies revealed that CYLD, an anti-inflammatory factor, is the direct target of miR-301-b. CYLD governed osteoclastogenesis through activating NF-κB signaling.

Investigations of osteoporosis on lncRNA or circRNA were limited in vivo. LncRNA Nron is highly enriched in bone tissue and markedly decreased in OVX mice [177]. Osteoclasts isolated from OVX mice or osteoclast-specific Nron knockout mice (*Nron*^*flox/flox*^; *Ctsk-Cre* mice) also expressed lower *Nron* with higher bone resorption. On the contrary, osteoclast-specific Nron overexpression transgenic mice (using Ctsk promoter) or pharmacological overexpression of Nron developed higher bone mass owing to suppressed osteoclastogenesis. Mechanistically, Nron interacted with CUL4B via its functional motif NCM2 to regulate the Erα pathway through ubiquitination in osteoclasts (Fig. 5).

In summary, numerous studies have shown that ncRNAs are recognized as key regulators of osteoporosis. In this section, we mainly reviewed some lncRNAs and miRNAs with essential roles in osteogenesis and osteoclastogenesis in vivo and their potential molecular mechanisms and translational significance. Nevertheless, there are few studies investigating the gain- or loss-of-function of circRNAs in transgenic animals until the submission of the manuscript. NcRNA-targeted genes affect different signaling pathways, which are important in the regulation of bone formation and absorption. More research is needed to study the manipulation of ncRNA expression in the future, which will establish the pathogenesis of osteoporosis and provide a potential therapeutic strategy for osteoporosis. An in-depth understanding of the regulation of those ncRNAs will be instrumental in exploring explicit targets to diagnose and treat osteoporosis. Table 2 summarizes the role of some ncRNAs in osteoporosis and their mechanisms through bone absorption validated in the transgenic mice model.

**Table 2:** The role of some ncRNAs in osteoporosis and their mechanisms through bone absorption validated in the transgenic mice model

ncRNA type	ncRNA name	Target genes and pathways	Transgenic mice	Function	Ref
miRNA	miR-21	Spry1 and PDCD4	miR-21-/-	miR-21 deficiency inhibited bone resorption and osteoclast function	[173]
miRNA	miR-128	SIRT1/NF-κB signaling	miR-128 fl/fl; LysM-Cre	Osteoclastic deletion of miR-128 suppressed osteoclastogenesis and exerted a protective effect against bone loss	[174]
miRNA	miR-143/145	Cd226 and Srgap2	miR-143/145-/-	miR-143/145 were shuttled into osteoclasts in extracellular vesicles and triggered osteoclastic activity	[169]
miRNA	miR-146a	RANKL/OPG signaling	miR-146a-/-	OC activities were impaired in the miR-146a KO mice exposed to estrogen deficiency	[123]
miRNA	miR-148a	NRP1	miR-148a-/-	miR-148a KO protects mice against excessive bone resorption	[175]
miRNA	miR-301b	CYLD/NF-κB signaling	miR-301-bfl/fl; LysM-Cre	Osteoclastic miR-301-b ablation inhibited OVX-induced osteoclastogenesis	[176]
lncRNA	Nron	CUL4B/Erα signaling	Nronfl/fl; Ctsk-Cre	Nron knockout mice exhibit an osteopenia phenotype with elevated bone resorption activity	[177]

## ncRNAs in osteoarthritis

Osteoarthritis (OA) is one of the prevalent diseases triggering pain and disability, which has a profound impact on the quality of life and socio-economic burden/cost throughout the world [178]. It usually affects joints in the hips, knees, and fingers, with characteristics of cartilage destruction, subchondral bone remodeling, and osteophyte formation. The incidence rate of OA has increased in recent years, affecting over 25% of the population over 18 years of age [179]. Most patients with OA have not received adequate treatment and can only alleviate symptoms. Identifying specific biomarkers for early diagnosis, subgroup classification, stage, and disease prognosis of OA is urgent [180]. A comprehensive understanding of molecular mechanisms of pathogenic OA will help discover new biomarkers so as to prevent or delay the deterioration of the disease [179]. It is well-known that ncRNAs can act as epigenetic regulators in cartilage formation and homeostasis by mediating the proliferation, differentiation, and ECM biosynthesis of chondrocytes [180]. The abnormal expression of ncRNAs may result in ECM degradation, chondrocyte hypertrophy, and chondrocyte apoptosis and subsequently lead to OA. A growing number of in vivo studies have highlighted the vital functions of ncRNAs in maintaining cartilage homeostasis.

### Positive miRNA regulators for articular cartilage homeostasis

Recent studies revealed that Sox9 could act as a critical transcription factor during cartilage formation and pathogenesis of OA [181, 182]. MiR-140 is a cartilage-specific ncRNA in mouse embryos and zebrafish, which is regulated by Sox9 [98]. MiR-140 global knockout mice showed short stature and lower body weight in the early stages [98]. However, these mice started to present OA-like pathology in the knee joints after 12 months old. Consistent with the aging-induced OA model, miR-140^−/−^ mice also exhibited accelerated articular cartilage injury in a surgical arthritis model. In addition, in an antigen injection-induced arthritis model, cartilage-specific miR-140 overexpression transgenic mice exhibited milder OA symptoms. DNA array analysis, together with further in vitro studies and in vivo studies, validated that miR-140 directly inhibited Adamts-5 expression from postponing aggrecan degradation and OA progression. MiR-455, an intensely expressed ncRNA in human and mouse chondrocytes, could also maintain cartilage homeostasis governed by Sox9 [183, 184]. MiR-455-3p^−/−^ mice presented accelerated cartilage degeneration at both 5 months and 12 months old [184]. Consistent with this, the knee joints of miR-455^−/−^ mice developed OA-like pathology at 6 months of age. Administration of miR-455-3p and miR-455-5p mimics into the knee cartilage of mice subjected to DMM surgery significantly inhibited cartilage degeneration. HIF-2α, encoded by *EPAS1*, was validated as a target of miR-455-5p and − 3p. HIF-2α was a catabolic factor for maintaining cartilage homeostasis, knockdown of which using siRNA in vivo injection into the knee cartilage of miR-455^−/−^ mice dramatically alleviated the cartilage degeneration.

Runx2 is a dominant regulator of chondrocyte hypertrophy and OA progression, overexpression of which leads to hypertrophic differentiation of normal chondrocytes [185, 186]. MiR-204 and miR-211 are homologous miRNAs, maintaining joint homeostasis via a Runx2-dependent manner to inhibit OA pathogenesis [157]. Global knockout of miR204/211 (miR-204^fl/fl^; miR-211^fl/fl^; CMV-Cre mice) resulted in spontaneous OA at the age of 15 weeks. Mesenchymal progenitor conditional knockout of miR204/211 (miR-204^fl/fl^; miR-211^fl/fl^; Prx1-Cre mice) presented OA-like phonotypes gradually with age. Depletion of miR-204/211 in mesenchymal tissues led to Runx2 accumulation and catabolic activities in articular cartilage degradation. Additionally, mesenchymal progenitor cells in dKO mice produced excessive mesenchymal cells by activating the Ngf/Akt pathway and contributed to synovial hyperplasia. Strikingly, these OA phenotypes could be ameliorated by intra-articular injection of AAV5-miR-204 in OA mice or knocking down Runx2 in mice (miR-204^fl/fl^; miR-211^fl/fl^; Runx2^fl/+^; Prx1-Cre). Table 3 summarizes the role of some ncRNAs in osteoarthritis and their mechanisms validated in the transgenic mice model.

**Table 3:** The role of some ncRNAs in osteoarthritis and their mechanisms validated in the transgenic mice model

ncRNA type	ncRNA name	Target genes and pathways	Transgenic mice	Function	Ref
miRNA	miR-455-5p and − 3p	EPAS1; HIF-2α Mmp3 Mmp13	miR-455-/-	miR-455 KO developed cartilage degeneration mimicking OA and elevated expression of cartilage degeneration-related genes	[183]
miRNA	miR-455-3p	PAK2; TGF-β signaling	miR-455-3p-/-	miR-455-3p inhibits cartilage degeneration	[184]
miRNA	miR-21-5p	Spry1; ERK-MAPK signaling	miR-21-5p-/-	Promoting the process of TMJOA	[188]
miRNA	miR-21-5p	FGF18	miR-21-5pfl/fl; Col2a1-CreER	The articular cartilage degradation of miR-21-5p conditional knockout mice was significantly alleviated in spontaneous destabilization of the medial meniscus models.	[187]
miRNA	miR-483-5p	Matn3 Timp2	pri-miR-483 TG mice	Intra-articular injection of lentivirus LV3-miR-483-5p or TG483 mice exhibited significant acceleration and increased severity of OA	[192]
miRNA	miR-483-5p	mTORC1-HDAC4‐miR‐483‐5p pathway	TSC1f/f; Col2a1-Cre pri-miR-483 TG mice	N.A	[193]
miRNA	miR-34a-5p		miR-34a-/-	miR-34a-KO mice exhibited protection against DMM-induced cartilage damage	[205]
miRNA	miR-204/211	Runx2; Akt signaling	miR-204 fl/fl; miR-211 fl/fl; Prx1-Cre or CMV-Cre	Maintaining healthy homeostasis of mesenchymal joint cells to counteract OA pathogenesis	[157]
miRNA	miR-146a	Camk2d Ppp3r2	miR-146a-/-	miR-146a KO mice was alleviated in spontaneous and instability-induced OA models	[191]
miRNA	miR-140	Adamts-5	miR-140-/-	Loss of miR-140 contributes to the development of age-related OA-like changes	[98]
miRNA	miR-141/200c	SIRT1; IL-6/STAT3	miR-141/200cfl/fl; Col2a1-CreERT2	Increased retention of NPs inside joint space in cKO mice	[189]

### Negative miRNA regulators for cartilage homeostasis

Utilizing Gene Expression Omnibus (GEO) datasets and further independent cohort and molecular biological validation, Wang and coworkers identified that miR-21-5p was markedly upregulated in OA patients [187]. They subsequently generated cartilage-specific miR-21-5p knockout mice by crossing miR-21-5p^fl/fl^ and Col2a1-CreERT2 mice. The cKO mice inhibited mice spontaneous OA at the 12th month of age, and the destabilization of the medial meniscus (DMM) induced OA. Intra-articular administration of antagomiR-21-5p significantly ameliorated articular cartilage degradation in the mice DMM model, while agomiR-21-5p aggravated it. Further studies indicated that FGF18 was a direct target of miR-21-5p, which was validated in vivo that the protein level of FGF18 in cKO mice was increased. Mir-21-5p was also related to the temporomandibular joint osteoarthritis (TMJOA) [188]. In a unilateral anterior crossbite model, miR-21-5p^−/−^ mice alleviated the progression of TMJOA, expressing less inflammatory-related genes and proteins. MiRNA target databases with further experiments validated that *Spry1* was a target gene of miR-21-5p. MiR-141/200c was also found to be enhanced in OA patients identified by microarray and further independent cohort [189]. Cartilage conditional knockout of miR-141/200c using miR-141/200c^fl/fl^; Col2a1- CreERT2 mice alleviated either aging or surgery-induced OA. Intra-articular administration of miR-141/200c delivered by chondrocyte-specific nanoparticles execrated OA pathogenesis while corresponding miR-141/200c inhibitor reagent exhibited chondroprotective effects. Specifically, miR-141/200c targeted SIRT1, a deacetylase, thus activating IL-6/STAT3 signaling to aggravate OA development.

MiR-146a was reported to be a biomarker of OA, which was elevated in the articular cartilage of early OA patients [190] and mice subjected to DMM surgery [191]. MiR-146a^−/−^ mice suppressed both spontaneous OA and knee destabilization-induced OA [191]. Lenti-miR-146a-mimic inhibited cartilage matrix-related Sox9 and Col2a1 expression in the primary mouse articular chondrocytes, while Lenti-miR-146a-inhibitor imparted a reverse effect. This result was validated in the DMM-induced mice model, indicating that suppressing miR-146a has a potential therapeutic effect on OA. The regulation of miR-146a on cartilage anabolism was considered as miR-146a targeted several genes in vitro and in vivo through an NF-кB-dependent signaling, including *Tgif1*, *Camk2d*, and *Ppp3r2*. The expression of miR-483-5p was increased either in the articular cartilage of OA patients or DMM-induced OA mice [192]. Bai group generated doxycycline-inducible miR-483 transgenic mice to investigate the pivotal role of miR-483-5p on OA [192, 193]. These mice exhibited more severe symptoms both in DMM-induced OA and aging-induced OA. Particularly, miR-483-5p was responsible for the expression of mTORC1, which was HDAC4 dependent. In addition, miR-483-5p directly targeted Matn3 and Timp2 to promote chondrocyte hypertrophy, extra-cellular matrix degradation, and cartilage angiogenesis. MiR-34a-5p was a p53-mediated ncRNA that participated in various human diseases, including OA. MiR-34a-5p was remarkably elevated in the plasma, cartilage, and synovium of OA patients and DMM-induced OA mice, which was correlated to obesity. Intra-articular administration of miR-34a-5p antisense oligonucleotide or miR-34a-5p global knockout protected articular cartilage from DMM-induced damage with or without a high-fat diet. Using RNA-sequencing with bioinformatics analysis, *Pparg* was predicted as a target of miR-34a-5p, which still needs to be validated further.

### CircRNAs in osteoarthritis

Due to the rapid development of high-throughput RNA-sequencing methods, more than 30,000 circRNAs have been reported in the last few years [62]. The research on circRNAs in OA has also been constantly emerging. Expression of circNFKB1 [194] and circGCN1L1 [195] has been reported to be up-regulated in human chondrocytes via RNA sequencing. Knockdown of circNFKB1 inhibited ECM catabolism and promoted osteoarthritis progression through interacting with ENO1 and sustaining the NF-κB signaling pathway. Ad-circNFKB1 was further intra-articularly injected into DMM-induced OA mice to determine its role in vivo, and the results showed that circNFKB1 aggravated OA progression, as manifested by cartilage destruction and osteophyte formation. While circGCN1L1 facilitated chondrocyte apoptosis, induced inflammation in synoviocytes, and decreased the anabolism of ECM via sponging miR-330-3p and targeting TNF-α in TMJ OA. CircRNAs can also be protective for the progression of OA. For example, circSERPINE2 [196], circPDE4B [197], and circFOXO3 [198] were downregulated in OA cartilage. circSERPINE2 protected articular cartilage via targeting miR-1271 and E26 transformation-specific-related gene (ERG). It could alleviate chondrocyte apoptosis and promote anabolism of ECM through the miR-1271-ERG pathway. Furthermore, intra-articular injection of adeno-associated virus overexpressing circSERPINE2 alleviated OA phenotype in a rabbit model. CircPDE4B regulated chondrocyte cell proliferation and ECM metabolism; the circPDE4B–RIC8A axis played an important role in regulating downstream p38 MAPK signaling. circFOXO3 activated PI3K/AKT-mediated autophagy, further alleviating apoptosis of chondrocytes and promoting anabolism of the ECM. As cartilage destruction and osteophytes decreased in Lv-circFOXO3 DMM mice, the anti-OA effect of circFOXO3 in vivo was also validated. CircPARD3B was downregulated in synovial tissues in OA, and it inhibited synovial angiogenesis via targeting miR-326 and SIRT1 [199].

### LncRNAs in osteoarthritis

**Fig. 6:**
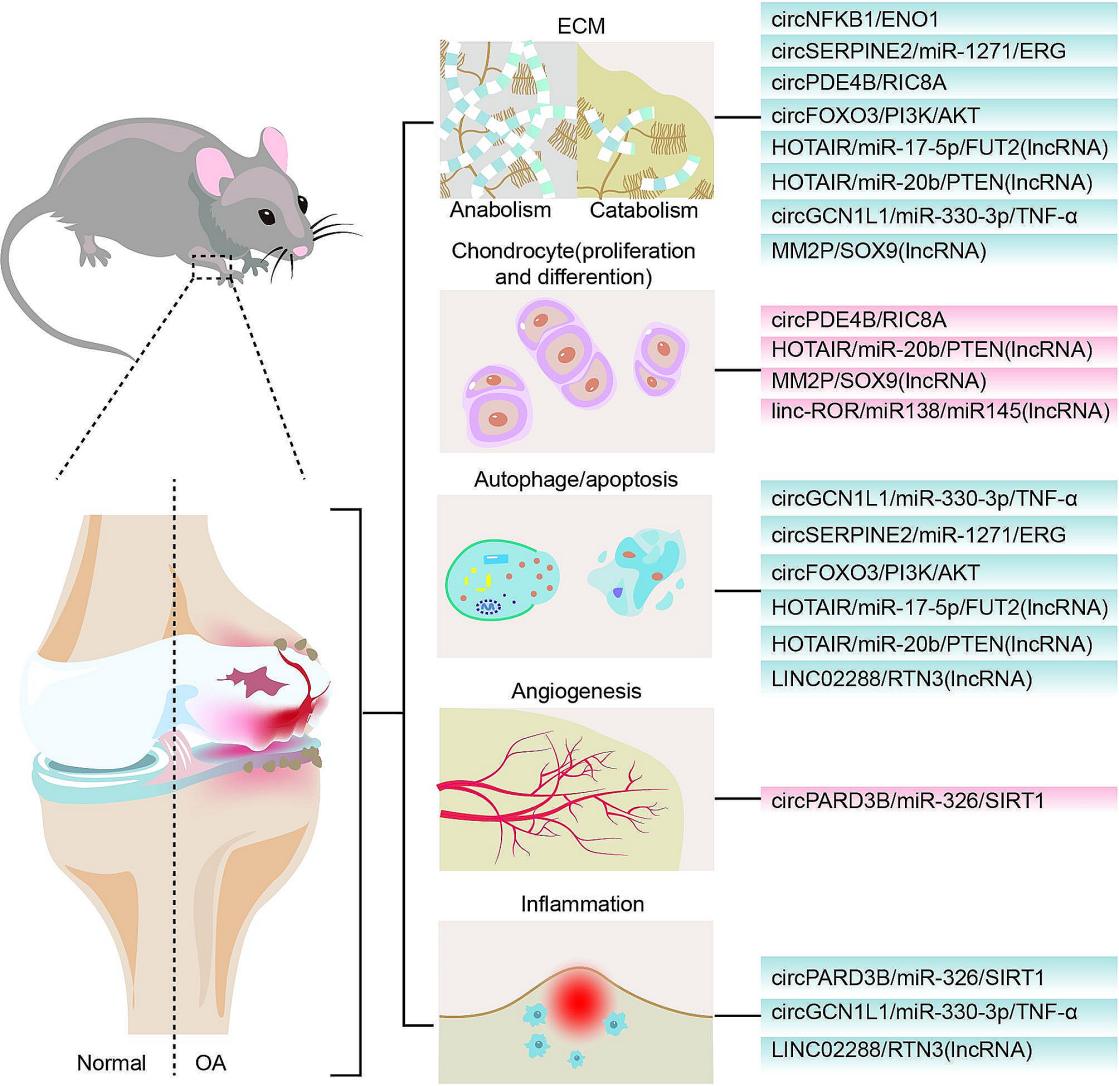
Illustration of the roles of ncRNAs that are experimentally proved in transgenic mice models as key regulators in the molecular mechanism of OA. Nc RNAs contribute to many pathologic processes in OA, including (**a**) ECM degradation, (**b**) chondrocyte proliferation and differentiation, (**c**) autophagy and apoptosis, (**d**) angiogenesis, and (**e**) inflammatory events

LncRNAs do not translate into polypeptides; however, they have essential effects on regulating the expression of specific genes. During the pathogenesis of OA, many lncRNAs have been discovered to be dysregulated in cartilage or synovial fluid. The function of lncRNAs in the pathogenesis of OA has been revealed with more details in recent research. HOTAIR [200] and LINC02288 [201] were found to be upregulated in human OA cartilage, and LINC02288 promoted chondrocyte apoptosis and inflammation by targeting the miR-347a-3p/RTN axis. HOTAIR directly bound to miR-17-5p and indirectly upregulated the expression of FUT2 and exacerbated chondrocyte apoptosis and ECM degradation. HOTAIR/miR-17-5p/FUT2 axis contributed to OA progression via the Wnt/β-catenin pathway. Intra-articular injection of the lentivirus expressing HOTAIR or FUT2 showed more severe damage to the cartilage. Another study also revealed that HOTAIR [202] promoted the progression of OA by inhibiting the proliferation of chondrocytes and promoting apoptosis and ECM degradation via regulating the miR-20b/PTEN axis. On the contrary, MM2P [203] was protective for the differentiation of chondrocytes, enhanced the expression of Colla2 and Acan and promoted the secretion of proteoglycan and type II collagen in chondrocytes by inducing M2 macrophage polarization, and accelerating the delivery of M2-derived exosomal SOX9 into chondrocytes. Another lncRNA, linc-ROR [204], also promoted mesenchymal stem cells chondrogenesis and cartilage formation via regulating SOX9 expression, linc-ROR functioned as a sponge for miR-138 and miR-145. They both suppressed BMSCs chondrogenesis activity and SOX9 expression, while co-expression of linc-ROR showed a rescuing effect. Figure 6 summarizes the roles of some ncRNAs that have been proved in transgenic mice models as regulators in the molecular mechanism of OA.

## Conclusions

Based on the evidence from hundreds of in vivo studies, it has been clearly demonstrated that ncRNAs play essential roles in skeletal development and diseases by regulating gene expression. The difficulty of mapping the interplay regulation networks of ncRNAs in the development and diseases of the skeletal system is identifying the critical ones among thousands of ncRNAs abundant in the skeletal tissues. Tissue-specific ncRNAs should be identified and might be critical in vivo systems. Thousands of in vitro studies are informative in terms of identifying the direct targets and related functions of each ncRNA in a specific cell type. However, the functional validation in vivo finally pins down the roles of specific ncRNAs in the physiological system.

## Data Availability

Not applicable.
